# Supplementary material for: ETV4 is a mechanical transducer linking cell crowding dynamics to lineage specification
Source: Nat Cell Biol. 2024 May 3;26(6):903–16. doi: 10.1038/s41556-024-01415-w (PMC11178500; doi:10.1038/s41556-024-01415-w)
Supplement: Supplementary file 1 — Reporting Summary [file 41556_2024_1415_MOESM1_ESM.pdf]

nature portfolio

Corresponding author(s): Jiwon Jang
Last updated by author(s): Mar 10, 2024

Reporting Summary

Nature Portfolio wishes to improve the reproducibility of the work that we publish. This form provides structure for consistency and transparency in reporting. For further information on Nature Portfolio policies, see our Editorial Policies and the Editorial Policy Checklist.

Statistics

For all statistical analyses, confirm that the following items are present in the figure legend, table legend, main text, or Methods section.

n/a	Confirmed	
☐	☒	The exact sample size (*n*) for each experimental group/condition, given as a discrete number and unit of measurement
☐	☒	A statement on whether measurements were taken from distinct samples or whether the same sample was measured repeatedly
☐	☒	The statistical test(s) used AND whether they are one- or two-sided<br>*Only common tests should be described solely by name; describe more complex techniques in the Methods section.*
☒	☐	A description of all covariates tested
☒	☐	A description of any assumptions or corrections, such as tests of normality and adjustment for multiple comparisons
☐	☒	A full description of the statistical parameters including central tendency (e.g. means) or other basic estimates (e.g. regression coefficient) AND variation (e.g. standard deviation) or associated estimates of uncertainty (e.g. confidence intervals)
☐	☒	For null hypothesis testing, the test statistic (e.g. *F*, *t*, *r*) with confidence intervals, effect sizes, degrees of freedom and *P* value noted<br>*Give P values as exact values whenever suitable.*
☒	☐	For Bayesian analysis, information on the choice of priors and Markov chain Monte Carlo settings
☒	☐	For hierarchical and complex designs, identification of the appropriate level for tests and full reporting of outcomes
☒	☐	Estimates of effect sizes (e.g. Cohen's *d*, Pearson's *r*), indicating how they were calculated

Our web collection on statistics for biologists contains articles on many of the points above.

Software and code

Policy information about availability of computer code

Data collection	Confocal images were taken by ZEISS LSM800 confocal microscope (ZEISS). Realtime PCR result were collected by CFX Connect Real-Time PCR Detection System (BIO-RAD). Western blot results were visualized with Amersham imager 680 (Amersham).
Data analysis	Sequencing data sets were processed and analyzed using the following tools:<br>HISAT v2.1.0<br>StringTie v2.1.3b<br>gProfiler ve109_eg56_p17_773ec798<br>GSEA v4.3.2<br>R v3.6.3<br>R package DESeq2 v1.38.3<br>R package DEsingle v1.18.1<br>Images were processed and analyzed using the following tools:<br>ImageJ v1.53<br>Statistical analysis was performed using the following tools:<br>GraphPad Prism v9.1.0<br>gel analysis was performed using the following tools:<br>Multiguage v3.0

For manuscripts utilizing custom algorithms or software that are central to the research but not yet described in published literature, software must be made available to editors and reviewers. We strongly encourage code deposition in a community repository (e.g. GitHub). See the Nature Portfolio guidelines for submitting code & software for further information.

Data

Policy information about availability of data

All manuscripts must include a data availability statement. This statement should provide the following information, where applicable:

- Accession codes, unique identifiers, or web links for publicly available datasets
- A description of any restrictions on data availability
- For clinical datasets or third party data, please ensure that the statement adheres to our policy

Data are available in the main text, supplementary materials, and Gene Expression Omnibus (GSE183702)

Human research participants

Policy information about studies involving human research participants and Sex and Gender in Research.

Reporting on sex and gender	H1 (male) and H9 (female) hESCs cell lines were used to cover all sex in this study.
Population characteristics	N/A
Recruitment	N/A
Ethics oversight	This work was approved by the Human Stem Cell Research Oversight Committee at Pohang University of Science and Technology (PIRB-2021-R035)

Note that full information on the approval of the study protocol must also be provided in the manuscript.

Field-specific reporting

Please select the one below that is the best fit for your research. If you are not sure, read the appropriate sections before making your selection.

☒ Life sciences ☐ Behavioural & social sciences ☐ Ecological, evolutionary & environmental sciences

For a reference copy of the document with all sections, see nature.com/documents/nr-reporting-summary-flat.pdf

Life sciences study design

All studies must disclose on these points even when the disclosure is negative.

Sample size	Preliminary experiments were performed when possible to determine requirements for sample size. Sample size sufficiency was determined by preliminary data or discussion. For statistical significance, the sample size was always independently performed three or more times(except for a few supplementary data with two independent experiments).
Data exclusions	No data were excluded from the analysis.
Replication	All experiments were replicated or performed independently for at least three times(except for a few supplementary data with two independent experiments).
Randomization	Randomization is not applicable to our study since all experiments were conducted on cultured cells. Any variations observed between treatment groups are not attributed to sampling bias.
Blinding	In general, all investigators were blind when they execute and gain data. During the experiments the investigators needed to know the media composition to maintain or induce differentiation of embryonic/pluripotent stem cells. Data analysis steps were blinded when available.

Reporting for specific materials, systems and methods

We require information from authors about some types of materials, experimental systems and methods used in many studies. Here, indicate whether each material, system or method listed is relevant to your study. If you are not sure if a list item applies to your research, read the appropriate section before selecting a response.

Materials & experimental systems

n/a	Involved in the study
☐	☒ Antibodies
☐	☒ Eukaryotic cell lines
☒	☐ Palaeontology and archaeology
☒	☐ Animals and other organisms
☒	☐ Clinical data
☒	☐ Dual use research of concern

Methods

n/a	Involved in the study
☒	☐ ChIP-seq
☒	☐ Flow cytometry
☒	☐ MRI-based neuroimaging

Antibodies

Antibodies used

Primary antibodies:
Mouse anti-Pax-6, Santa Cruz, SC-81649 (PAX6, monoclonal)
Mouse anti-Oct-3/4 (C-10), Santa Cruz, SC-5279 (C10, monoclonal)
Rabbit anti-SOX2, Millipore, AB5603 (Polyclonal)
Rat anti-beta1 integrin (CD29), BD, 553715 (9EG7, monoclonal)
Rabbit anti-ETV1, NOVUSBIO, NBP2-57731 (Polyclonal)
Rabbit anti-ETV4, Proteintech, 10684-1-AP (Polyclonal)
Rabbit anti-ETV5, Proteintech, 13011-1-AP (Polyclonal)
Rabbit anti-Ki-67 (D3B5), Cell Signaling Technology, 9129S (D3B5, monoclonal)
Rabbit anti-pFAK (D20B1), Cell Signaling Technology, 8556S (D20B1, monoclonal)
Rabbit anti-pERK [P-p44/42 MAPK(T202/Y204)], Cell Signaling Technology, 4370S (D13.14.4E, monoclonal)
Rabbit anti-pAkt, Cell Signaling Technology, 9271T (Polyclonal)
Rabbit anti-FGFR1 (D8E4), Cell Signaling Technology, 9740S (D8E4, monoclonal)
Rabbit anti-HA-Tag (C29F4), Cell Signaling Technology, 3724S (C29F4, monoclonal)
Rat anti-HA-Tag (3F10), Roche, 12158167001 (3F10, monoclonal)
Rabbit anti-pMLC, Cell Signaling Technology, 3674S (Polyclonal)
Goat anti-Nanog, R&D, AF1997 (Polyclonal)
Goat anti-Brachyury, R&D, AF2085 (Polyclonal)
Mouse anti-EEA1, BD, 610456 (14, monoclonal)
Rabbit anti-COP1, BETHYL, A300-894A (Polyclonal)
Rabbit anti-paxillin, NOVUSBIO, NBP2-57097 (Polyclonal)
Mouse anti-ZO1, ThermoFisher Scientific, 33-9100 (1A12, monoclonal)
Mouse anti-E-Cad, Cell Signaling Technology, 14472S (4A2, monoclonal)
Rabbit anti-MMP14, NOVUSBIO, NBP2-67415 (3-F7, monoclonal)
Rabbit anti-GFP, ThermoFisher Scientific, A11122 (Polyclonal)
Mouse anti-YAP, Santa Cruz, SC-101199 (63.7, monoclonal)
Rabbit anti-YAP/TAZ, Cell Signaling Technology, 8418S (D24E4, monoclonal)
mouse anti-β-actin, Santa Cruz, SC-47778 (C4, monoclonal)

Secondary antibodies:
anti-Rabbit IgG (H+L) Secondary Antibody, HRP, ThermoFisher Scientific, 31460
anti-Mouse IgG (H+L) Secondary Antibody, HRP, ThermoFisher Scientific, 31430
anti-Goat IgG (H+L) Cross-Adsorbed Secondary Antibody, Alexa Fluor 488, ThermoFisher Scientific, A-11055
anti-Mouse IgG (H+L) Highly Cross-Adsorbed Secondary Antibody, Alexa Fluor Plus 555, ThermoFisher Scientific, A-32773
anti-Mouse IgG (H+L) Highly Cross-Adsorbed Secondary Antibody, Alexa Fluor 647, ThermoFisher Scientific, A-31571
anti-Rabbit IgG (H+L) Highly Cross-Adsorbed Secondary Antibody, Alexa Fluor 488, ThermoFisher Scientific, A-21206
anti-Rabbit IgG (H+L) Highly Cross-Adsorbed Secondary Antibody, Alexa Fluor 555, ThermoFisher Scientific, A-31572
anti-Rat IgG (H+L) Cross-Adsorbed Secondary Antibody, Alexa Fluor 555, ThermoFisher Scientific, A-21434

Validation

- Mouse anti-Pax-6, Santa Cruz, SC-81649: The antibody guarantee covers the use of the antibody for WB, IP, IHC and IF applications. Species reactivity: Human, Mouse, Rat, Avian

- Mouse anti-Oct-3/4 (C-10), Santa Cruz, SC-5279: hESCs differentiation led to a reduced fluorescence signal shown by immunofluorescent staining (Data not included). The antibody guarantee covers the use of the antibody for WB and IF applications. No cross-reactivity may occur with Oct-3/4 isoform B. Species reactivity: Mouse, Rat and Human

- Rabbit anti-SOX2, Millipore, AB5603: hESCs differentiation led to a reduced fluorescence signal shown by immunofluorescent staining (Data not included). The antibody guarantee covers the use of the antibody for WB and IF applications. Species reactivity: Human, Mouse.

- Rat anti-beta1 integrin (CD29), BD, 553715: The antibody guarantee covers the use of the antibody for WB, IP, IHC and IF applications. Species reactivity: Mouse

- Rabbit anti-ETV1, NOVUSBIO, NBP2-57731: The antibody guarantee covers the use of the antibody for ICC and IF applications. Species reactivity: Mouse, Rat

- Rabbit anti-ETV4, Proteintech, 10684-1-AP: ETV4 Knockdown using two independent shRNAs led to a reduced fluorescence signal shown by immunofluorescent staining (Extended Data Fig. 4f). The antibody guarantee covers the use of the antibody for WB and IF applications. Species reactivity: Human, Mouse, Rat

- Rabbit anti-ETV5, Proteintech, 13011-1-AP: The antibody guarantee covers the use of the antibody for WB and IF applications. Species reactivity: Human, Mouse

- Rabbit anti-Ki-67 (D3B5), Cell Signaling Technology, 9129S: The antibody guarantee covers the use of the antibody for ICC and Flow cytometry applications. Species reactivity: Human, Mouse, Rat

- Rabbit anti-pFAK (D20B1), Cell Signaling Technology, 8556S: FAK inhibition using chemical(PND1186) led to a reduced fluorescence signal shown by immunofluorescent staining (Extended Data Fig. 9c).The antibody guarantee covers the use of the antibody for WB and IP applications. Species reactivity: Human

- Rabbit anti-pERK [P-p44/42 MAPK(T202/Y204)], Cell Signaling Technology, 4370S: The antibody guarantee covers the use of the antibody for WB, IP, IHC, IF, and Flow cytometry applications. Species reactivity: Human, Mouse, Rat, Hamster, Monkey, Mink, D. melanogaster, Zebrafish, Bovine, Dog, Pig, S. cerevisiae

- Rabbit anti-pAkt, Cell Signaling Technology, 9271T: The antibody guarantee covers the use of the antibody for WB, IP, IHC, IF, and Flow cytometry applications. Species reactivity: Human, Mouse, Rat, Hamster, Monkey, D. melanogaster, Bovine, Dog

- Rabbit anti-FGFR1 (D8E4), Cell Signaling Technology, 9740S: FGFR1 Knockdown using two independent shRNAs led to a reduced fluorescence signal shown by immunofluorescent staining (Extended Data Fig. 8c). The antibody guarantee covers the use of the antibody for WB, IP, IHC, IF, and Flow cytometry applications. Species reactivity: Human, Mouse, Rat, Monkey

- Rabbit anti-HA-Tag (C29F4), Cell Signaling Technology, 3724S: The antibody guarantee covers the use of the antibody for WB, IP, IHC, IF, flow cytometry and ChIP applications. Species reactivity: All Species Expected

- Rat anti-HA-Tag (3F10), Roche, 12158167001: The antibody was validated by staining H9 hESCs expressing HA ETV4(Extended Data Fig. 4d). The antibody guarantee covers the use of the antibody for WB, IF and ELISA applications. Species reactivity: Human

- Rabbit anti-pMLC, Cell Signaling Technology, 3674S: The antibody guarantee covers the use of the antibody for WB applications. Species reactivity: Human, Mouse

- Goat anti-Nanog, R&D, AF1997: hESCs differetiation led to a reduced fluorescence signal shown by immunofluorescent staining (Data not included). The antibody guarantee covers the use of the antibody for WB and IF applications. Species reactivity: Human

- Goat anti-Brachyury, R&D, AF2085: The antibody guarantee covers the use of the antibody for WB, IHC, IF and ChIP applications. Species reactivity: Human, Mouse

- Mouse anti-EEA1, BD, 610456: The antibody guarantee covers the use of the antibody for WB, IHC, IF and IP applications. Species reactivity: Human, Rat, Chicken, Dog

- Rabbit anti-COP1, BETHYL, A300-894A: COP1 Knockdown using shRNA led to a reduced fluorescence signal shown by immunofluorescent staining (Extended Data Fig. 7c). The antibody guarantee covers the use of the antibody for WB and IP applications. Species reactivity: Human, Mouse

- Rabbit anti-paxillin, NOVUSBIO, NBP2-57097: The antibody guarantee covers the use of the antibody for WB, ICC and IF applications. Species reactivity: Mouse, Rat

-Mouse anti-ZO1, ThermoFisher Scientific, 33-9100 (monoclonal): The antibody guarantee covers the use of the antibody for WB, IHC, IF, ICC, Flow, ELISA and IP applications. Species reactivity: Human, Mouse, Dog, Rhesus monkey

- Mouse anti-E-Cad, Cell Signaling Technology, 14472S: The antibody guarantee covers the use of the antibody for WB, IP, IHC, IF and Flow cytometry applications. Species reactivity: Human, Mouse, Rat

- Rabbit anti-MMP14, NOVUSBIO, NBP2-67415: The antibody was validated by staining H9 hESCs expressing MMP14 (Extended Data Fig. 5k). The antibody guarantee covers the use of the antibody for WB, IF, IHC and IP applications. Species reactivity: Human, Mouse, Rat

- Rabbit anti-GFP, ThermoFisher Scientific, A11122: The antibody guarantee covers the use of the antibody for WB, IHC, IP and ChIP applications. Species reactivity: Tag

- Mouse anti-YAP, Santa Cruz, SC-101199: COP1 Knockdown using shRNA led to a reduced fluorescence signal shown by immunofluorescent staining (Extended Data Fig. 3i). The antibody guarantee covers the use of the antibody for WB, IP, IF, IHC and ELISA applications. Species reactivity: Human, Mouse, Rat

- Rabbit anti-YAP/TAZ, Cell Signaling Technology, 8418S: The antibody guarantee covers the use of the antibody for WB and IP applications. Species reactivity: Human, Mouse, Monkey

- mouse anti-β-actin, Santa Cruz, SC-47778: The antibody guarantee covers the use of the antibody for WB, IP, IF and ELISA. Species reactivity: mouse, rat, human, avian, bovine, canine, porcine, rabbit, Dictyostelium discoideum and Physarum polycephalum. Cross-reactivity may occur with all six known isoforms of Actin in higher vertebrates (including cytoplasmic β- and γ- Actin isoforms, skeletal, cardiac, and vascular α-Actin isoforms, and enteric γ-Actin isoform).

Eukaryotic cell lines

Policy information about cell lines and Sex and Gender in Research

Cell line source(s)	H1 and H9 hESCs were purchased from WiCell.<br><br>HEK293T, ARPE19 and MCF-7 were purchased from ATCC.
Authentication	Cell lines were authenticated by short tandem repeat analysis and/or in vitro differentiation.
Mycoplasma contamination	Mycoplasma contamination was routinely checked and negative results were obtained.
Commonly misidentified lines (See ICLAC register)	No commonly misidentified cell lines were used in the study.
