# Supplementary figures and images for: ETV4 is a mechanical transducer linking cell crowding dynamics to lineage specification
Source: Nat Cell Biol. 2024 May 3;26(6):903–16. doi: 10.1038/s41556-024-01415-w (PMC11178500; doi:10.1038/s41556-024-01415-w)

**Fig. 1e**

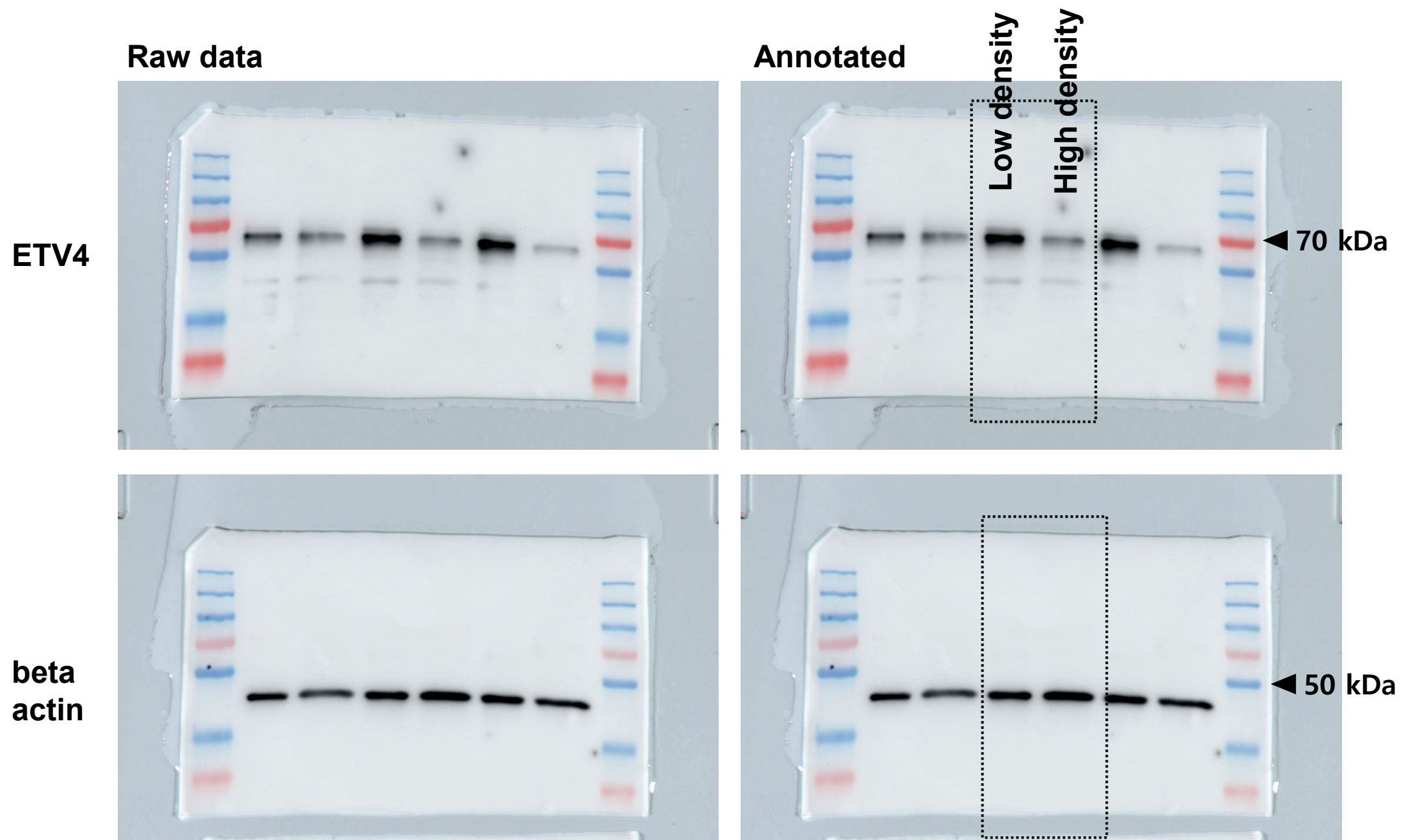

**Fig. 4e**

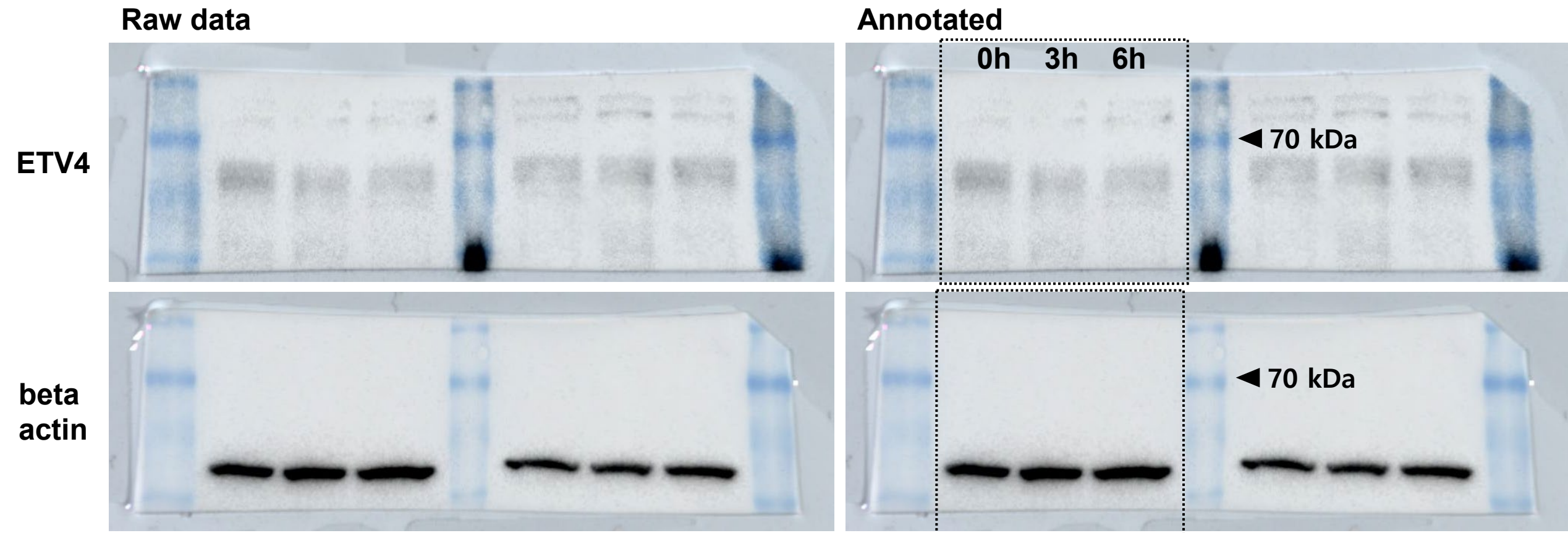

**Fig. 4e**

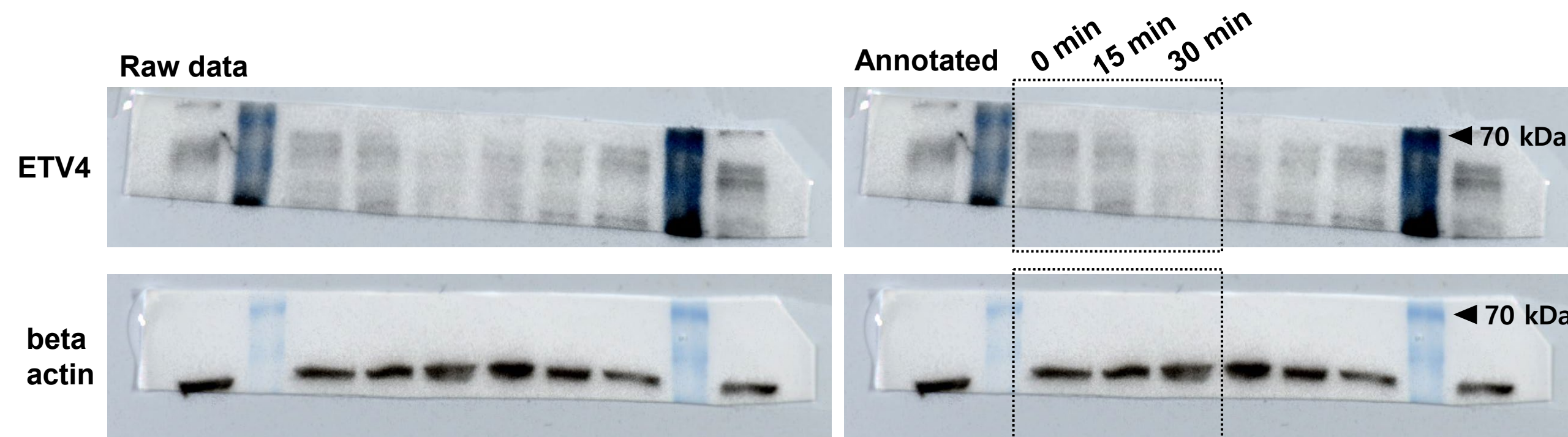

Supplement: Supplementary file 13 — Full-length, unprocessed gels. [file 41556_2024_1415_MOESM13_ESM.pdf]

**Fig. 4c**

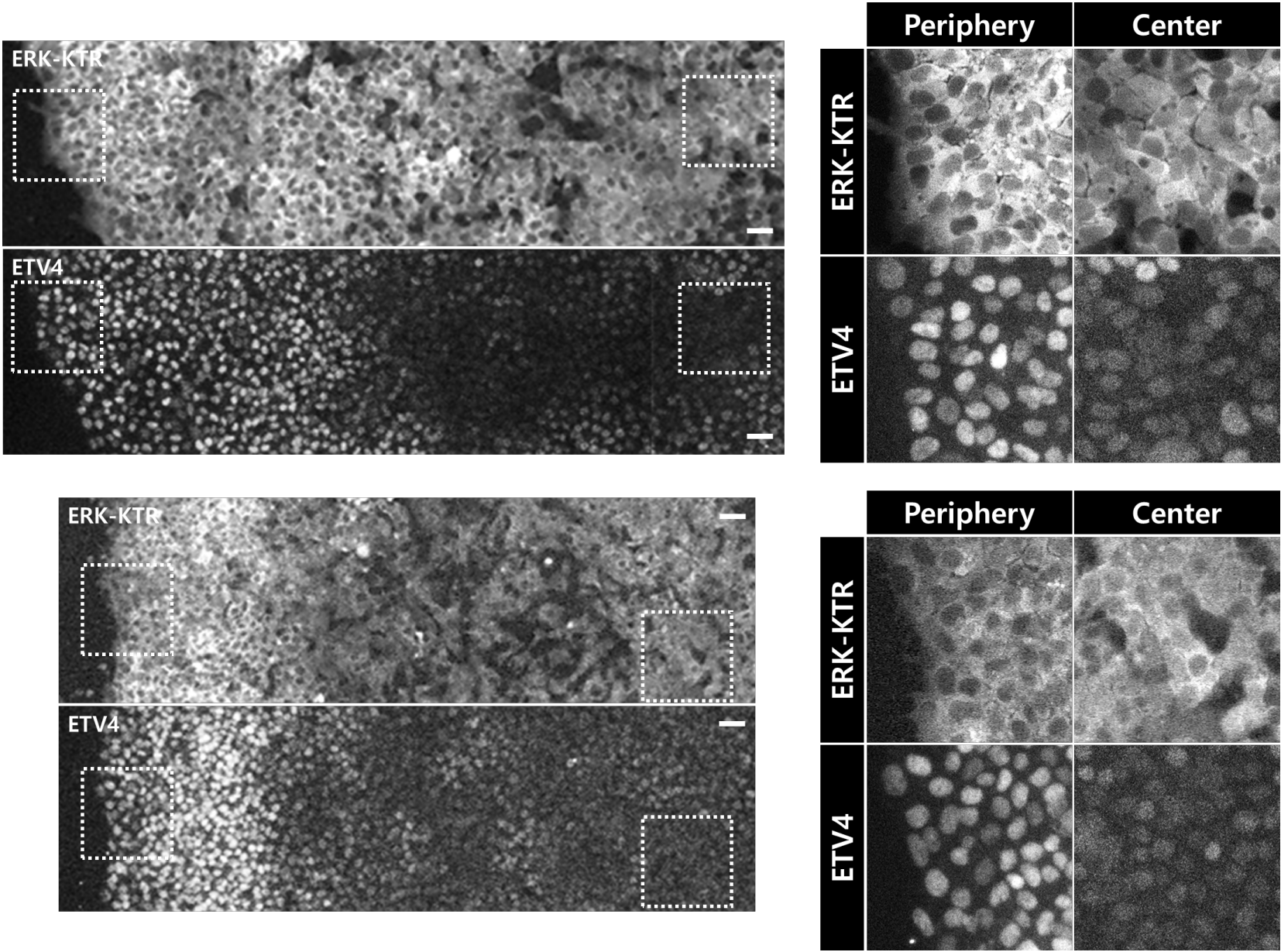

Scale bar: 25μm

Extended Data Fig. 6b

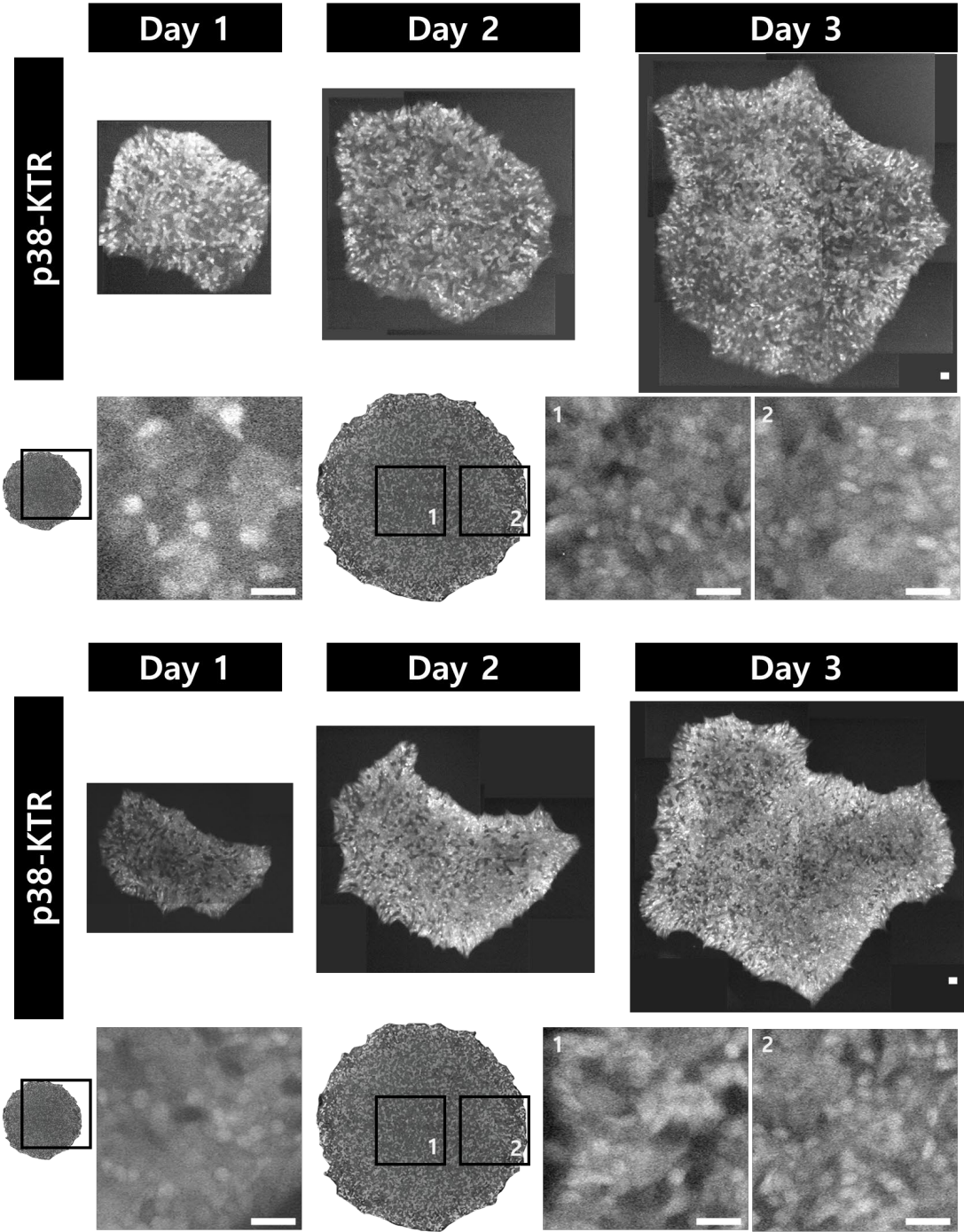

Extended Data Fig. 6c

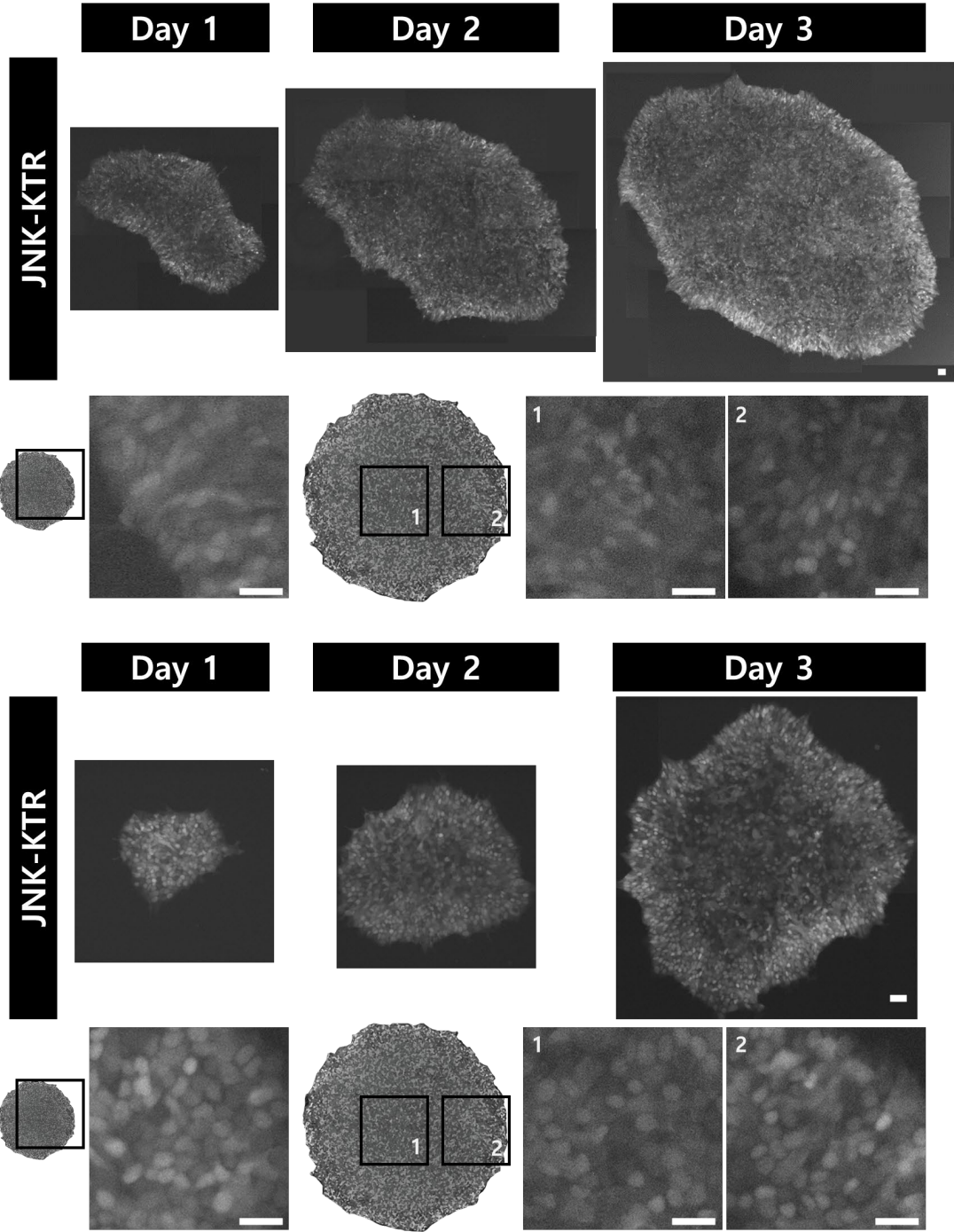

Supplement: Supplementary file 14 — Additional microscope images. [file 41556_2024_1415_MOESM14_ESM.pdf]
